# The body as a mirror of inequality in México during the second half of the twentieth century

**DOI:** 10.1590/S0104-59702024000100018en

**Published:** 2024-06-10

**Authors:** Moramay Lopez-Alonso

**Affiliations:** i Associate Professor, Department of History/Rice University. Houston – Texas – USA. moramay@rice.edu

**Keywords:** History, Stature, México, Nutrition, Living standards, Historia, Estatura, México, Nutrición, Estándares de vida

## Abstract

This paper examines how variations in the height and health of Mexicans during the second half of the twentieth century reflect the evolution of economic inequality, as its effects have repercussions on the health and nutritional conditions of the population. The average height of Mexican adults had a modest increase with respect to the possibilities of human plasticity. These anthropometric variations were the result of the incorporation of advances in science and technology leading to improved standards of living among the population. Body changes were impacted by dietary habits, urbanization, and government policies supporting food production and distribution.

## Resumen

Este trabajo examina cómo las variaciones en la estatura y la salud de los mexicanos durante la segunda mitad del siglo XX son un reflejo de la evolución de la desigualdad económica pues sus efectos repercuten en las condiciones de salud y alimentación de la población. La estatura promedio de adultos mexicanos tuvo un aumento modesto con respecto a las posibilidades de la plasticidad humana. Estas variaciones antropométricas fueron resultado de la incorporación de avances en ciencia y tecnología conducentes a mejorar los niveles de vida de la población. Los cambios corporales fueron impactados por hábitos alimenticios, de urbanización y políticas gubernamentales de apoyo a la producción y distribución de alimentos.

This paper discusses that in México there has been an improvement in the biological standard of living of the population in the last century, but these improvements are limited with respect to the possibilities of human plasticity, both in terms of the possibilities of scientific and technological advances made available since the twentieth century. They are also small when compared to economic growth cycles. During the period covered by our study, public policies were implemented that sought to generally improve the population’s standard of living. The emphasis and design of each policy changed during each six-year term, according to the objectives of the national development plan established by the president in office. This influenced the attention given to health and nutrition. This paper attempts to show that political decisions had an impact on the population’s biological standard of living. Research in the areas of nutrition and nutritional science progressed during this period in order to better understand the population, their eating habits, their state of health, and the changes they were undergoing as a result of the transformation the country was experiencing. However, this information was not always used as a basis for decision-making regarding food and health policies. As we will see in this paper, food production and distribution at the national level did not consider food quality. Although government advertising campaigns were created to publicize food distribution programs that included a component of the importance of good nutrition, these campaigns were overshadowed by the marketing of large private industrialized food companies. This paper seeks to contribute to the study of living standards and inequality based on the fields of public health history and economic history. The remainder of this paper is organized thematically. The following section discusses the evolution of anthropometric measures and their relevance for the analysis of standards of living. The third section will place this analysis in the context of the history of nutritional science, pathologies, public health policies, and food safety. The fourth part will provide the conclusions.

## Body changes, historical anthropometry, and inequality

For at least a century, physical anthropologists have been systematically collecting the heights of different populations in order to study their living conditions. The joint study of anthropology with human biology and auxology has allowed us to understand how nutrition, quality of health, and environment determine the final adult height of an individual as much as their genetic inheritance (Tanner, 1978; McKeown, 1976; Steckel, 1995, 2008). At the beginning of the century, Franz Boas began to collect anthropometric measurements in his physical anthropology studies that included height in order to substantiate his argument that differences between races were not immutable and that the environment could modify physical traits such as height and skull circumference (Steckel, 1995, p.1907). By the middle of the twentieth century, it was possible to assemble large databases based on the tools developed by statistics that were able to establish that the heights of human populations had a normal distribution.

During the twentieth century, some physicians studied the relationship between social differentiation and its effects on growth and maturation. J.M. Tanner is perhaps the best-known researcher on the subject due to his compilation of works at a global level (Eveleth, Tanner, 1990; Tanner, 1981). For Tanner, the body is a mirror of society (Bogin, 2001, p.240). His research findings indicated that stunted growth and maturation of children and adolescents tended to occur in social groups with unfavorable economic conditions. A key role in this is played by human plasticity, which is the physiological process of the physical growth forms of each individual (Bogin, Loucky, 1997, p.17). Human growth depends on multiple genetic and environmental factors. The provision of nutrition and sanitation and disease prevalence are often considered to be the environmental factors that most influence the plasticity of human growth (Niere et al., 20 mayo 2020, p.1). As we will see below, this framework of analysis will allow us to interpret the determinants of the evolution of the height of the Mexican population in light of diet quality, exposure to disease and physical activity, and work for growth during the first 20 years of life (Steckel, 1995, p.1910).

In the mid-twentieth century, economists and other social scientists began to consider anthropometric measures as a variable for assessing the standard of living of the population. This was the time when methodologies for measuring human development indexes were beginning to be developed by international organizations. The dark legacy of Nazi Germany’s racist policies meant that human height was not included as a variable in calculating the human development index. However, economists began to study the correlation between average height and income levels and differences in income distribution (Steckel, 1995, p.1908). Scholars in the field of development economics examined how the evolution of the average heights of adult populations was a good standard of living indicator, especially in countries where governments did not have the resources to collect data or where a large part of the economically active population worked in the informal sector of the economy (Steckel, 2008, p.129).

Economists working on the design of public policies to promote social development became interested in the use of anthropometric measures to better understand the impact on the welfare levels of a population when the economic situation improves or worsens. This is because the average adult height reflects the net results of the quality of health and nutrition that individuals had during their first 20 years of life. This makes it possible to know the results of an investment in health or nutrition. Anthropometric measures were considered a useful tool for evaluating the effectiveness of welfare programs that could be used by both development economists focused on the design and evaluation of public policies and economic historians (Steckel, 1995, p.1911). Both fields of study shared an interest in understanding long-term economic growth processes. These works were developed in the North American academy. However, it is necessary to emphasize that it was the French historians of the *Annales* School, in the mid-twentieth century, who were pioneers in creating measurements to study the evolution of long-term standards of living.

Regarding how to read the evolution of human height as an indicator of levels of well-being, it is important to clarify that the final height of an adult is the result of the quality of health and nutrition from the moment of conception and during the first two decades of life. On an individual scale, the genetic component is important in determining the height of an individual, but in the aggregate, this component loses relevance. Anthropometric studies examine changes in population means. Population heights have a normal distribution. There are also differences between male and female adult heights due to sexual dimorphism. This means that in a given population, living under normal circumstances, females are 12 to 13 centimeters shorter than males. This is a consequence of the different timing of the adolescent growth spurt, which gives boys a couple of extra years before the adolescent growth spurt begins (Bogin, 2001).

However, each population has its specificities given its demographic and geographic characteristics and its general history. Let us now examine the Mexican case in light of this framework of analysis. Previous studies have examined the evolution of living standards in México for the population born between 1850 and 1950 (López-Alonso, 2007, 2012). This period of study does not follow the chronology normally used in traditional historiography that follows the main political events but contemplates several processes relevant to the evolution of standards of living. The traditional historiography of the national period begins in 1821 with the promulgation of independence. The first five decades were characterized by political instability caused by civil wars and foreign invasions. This was followed by the government of Porfirio Díaz, which lasted 35 years (1876-1911). The government, which was described as a dictatorship, ended with the outbreak of the Revolution, whose worst armed conflict lasted ten years. Although the unrest continued until the early 1930s, the post-revolutionary period is counted from 1920 onwards.

Previous anthropometric studies use a different chronology. These studies cover a century (1850-1950) that focuses on demographic, sanitary, scientific, technological, and productive process transformations. These changes include the beginning of the industrialization process in 1876. There is also a demographic transition caused by a process of urbanization that began in the final decades of the nineteenth century and accelerated after the Revolution (1910-1920). The adoption of germ theory, investments in sanitary infrastructure in the most populated cities, and national childhood vaccination campaigns were elements that contributed to improving the quality of life and increasing life expectancy in the early decades of the twentieth century. These transformations can be seen in long-term studies and are not always altered by political events. In fact, the findings of studies covering the period 1850-1950 show that the evolution of living standards did not always move in the same direction as economic growth cycles, nor did it have the same trajectory for the entire population; in Mexico, in this period, there was divergence in regional and socioeconomic levels. Regional divergence showed that populations in the Northern and North-Central (Bajio) states experienced an improvement in biological living standards as they had a higher average height than their South-Central counterparts. The divergence in socioeconomic levels shows that the biological standards of living of the middle and upper classes followed a pattern of increase in height similar to that of populations in high-income European countries, or the United States, while the lower classes had a stagnation (López-Alonso, 2012, p.207). This calls into question the assertions of the official history, written in the first decades of the post-revolutionary period (post-1920), which argued that the living standards of the majority of the population during the Porfirio Díaz presidency (1876-1911) had been deteriorating until the outbreak of the Revolution in 1910 and that, after the Revolution, those who had less improved their standard of living. The results of the height analysis invite us to qualify these statements by showing that living standards had been worsening since the 1850s and that, during Porfirio’s term in office, there were periods of improvement and deterioration. Similarly, the improvement in living standards did not begin to be observed until the late 1930s with the implementation of welfare policies by President Lázaro Cárdenas (1934-1940). Results varied by region and social class. From anthropometric history studies, it can be concluded that in México, populations born before 1950 had an evolution in height determined by their region of origin and, above all, by their level of income.

For the second decade of the twentieth century, it is possible to conduct studies that include more detailed information on socioeconomic and health determinants because there are better data sources. Therefore, the previous works were completed in 1950. It is also important to see the political context under what circumstances and for what purpose the data sources were created to understand their scope and limitations. During the second half of the twentieth century, it can be said that there was relative political stability during which the country was governed by eight presidents from the same party: the Institutional Revolutionary Party (Partido Revolucionario Institucional, PRI). However, the economy had a mixed performance. The first decades (1950-1970) were characterized by relative economic stability, while in the following decades, there were several periods of crisis. At the same time, the population became mostly urban, with very high population growth during the first three decades and moderate growth towards the mid-1980s. Life expectancy at birth in 1950 was 49.7 years and 73.9 years in 2000, and in the same period, illiteracy levels decreased from 43.2% to 9.5%.

Starting in 1950, studies began to be compiled at the national level that allowed for a more detailed analysis of the indicators that can be used to measure the evolution of living standards. Although national population censuses were conducted approximately every decade, it was recognized that more complete studies on health and nutrition could yield information that would help to better diagnose the needs of the population. The federal government began to allocate resources to create research centers that could generate these types of studies. Thus, in the six-year term of Adolfo López Mateos (1958-1964), the first nutritional surveys were carried out in México (1958-1962). However, the resources allocated to the studies depended on the economic situation during the current administration and the objectives of the respective national development plan, as we will see below. For this reason, it was not until 1979 that there was another similar initiative: the food survey. In the following years, there were health surveys but without a focus on nutrition. At the beginning of the twenty-first century, the National Health Survey System conducted the National Health Survey 2000, which was the first nationwide study and applied a methodology that made it possible to include information on nutrition, health, height, and others with unprecedented coverage. The 2000 National Health Survey (México, 2000) and the 2006 National Health and Nutrition Survey (México, 2006) contain data that allow an evaluation of the impact of the social development programs of the final decades of the twentieth century, which were focused on improving living standards, as they collected information on height, health status, and nutrition. Although the questions in each survey were not identical, it is possible to decipher the correlation in three changes: lifestyles, eating habits, health, and height. There are also local and regional studies that give us a more precise vision of the living standards of a population and the correlation in height and quality of health and nutrition that help us to have a better understanding of the complex processes, in which the dynamics of the evolution of living standards takes place in an environment of economic growth with inequality (Sandoval, 1985; Cahuich Campos, 2001; Ramos Rodríguez, Sandoval Mendoza, 2007).

The analysis of the above-mentioned sources allows us to study the impact of political, economic, and demographic changes in México during this period on biological standards of living. Changes in dietary habits can be evaluated in terms of their impact on the population’s standard of living. This is part of the rapid urbanization process that led to changes in lifestyles which, in turn, modified the population’s diet and social and cultural practices linked to food.

## Mexican bodies in the second half of the twentieth century

**Figure 1:**
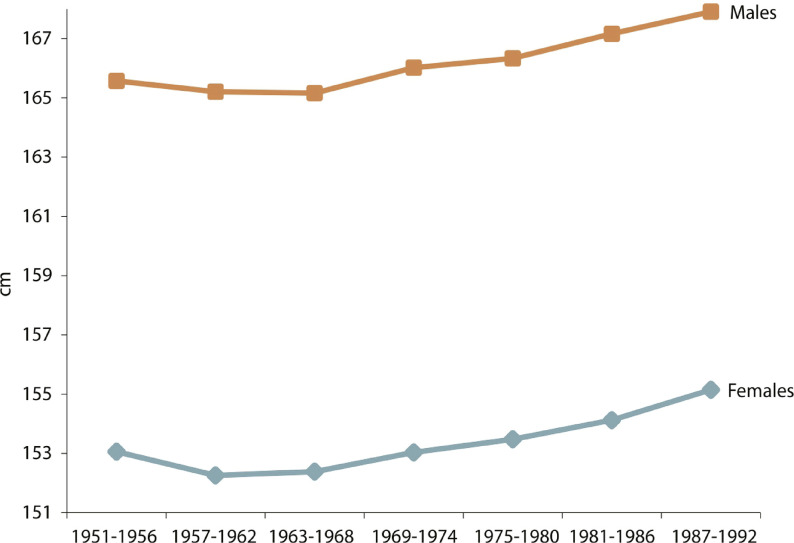
: Graph of estimated national heights (in cm) by 6-year cohorts, México, 1951-1992 (México, 2000, 2006)

It is possible to examine the evolution of the biological standards of living of Mexican men and women at the national level born during the second half of the twentieth century from data collected in the ENSA 2000 and ENSANUT 2006 national health surveys. These surveys contain data with which to build a basis for individuals born between 1951 and 1986. Following the norm of historical studies, we consider the heights of individuals between 20 and 49 years of age, since final height is reached at approximately 20 years of age, and after 50 years of age height may begin to decrease, depending on the quality of life and physical deterioration that took place before reaching that age. Since the data to construct the graph in Figure 1 come from two different surveys, an equality test comparing a cohort of 16-year-olds born between 1960 and 1976 was performed to find out whether the two samples were compatible. The samples were compatible with very small height differences (4 millimeters for males and 2.6 millimeters for females (López-Alonso, Vélez-Grajales, 2015, 2016, 2019). It was decided to use ENSA 2000 for cohorts born between 1951 and 1956. Both surveys are nationally representative. Table 1 shows the number of observations in each 6-year cohort.

**Table 1:** : Sample sizes by 6-year cohorts

**Cohort**	**Sex**	
	**Men**	**Women**
1951-1956	11,516	2,992
1957-1962	1,467	2,119
1963-1968	1,967	3,096
1969-1974	2,081	3,638
1975-1980	1,652	3,217
1981-1986	1,787	2,594
**Total**	**20,470**	**17,656**

Source: México (2000, 2006).

The evolution of stature was estimated using a simple linear regression model. Dummies were used to control for birth cohorts; school achievement was used as a proxy for socioeconomic status, and place of residence was used to examine the potential advantages or disadvantages of living in rural or urban settings (urban/rural penalty). The regression results showed that there was a slight decrease in height for individuals born in the 1950s, followed by an increase in subsequent decades. Figure 1 shows that trends for men and women are similar. The overall increase in height is about 2cm for women and 2.5cm for men. The results of the regressions in Table 2 show that men and women residing in rural areas are shorter in height than their counterparts in cities. Men and women with more schooling tend to be taller.

**Table 2:** : Height regression results for men and women

**Birth Cohort**	Women*	Men*
1951-1956 (omitted)	–	–
1957-1962	-0.389*** (0.195)	-0.199 (0.289)
1953-1968	-0.95 (0.178)	0.098 (0.273)
1969-1974	0.012 (0.174)	0.540** (0.273)
1975-1980	0.159 (0.181)	0.906*** (0.284)
1981-1986	0.728*** (0.191)	1.546*** (0.284)
**Education**		
Primary incomplete	1.684*** (80.24)	1.000** (0.419)
Primary complete	3.223*** (0.234)	1.963*** (0.406)
Secondary	4.674*** (0.24)	3.431*** (0.407)
Preparatory	6.004*** (0.255)	4.991*** (0.425)
Licenciate	7.616*** (0.318)	6.105*** (0.467)
Posgraduate	8.591*** (80.749)	6.983*** (0.906)
No schooling (omitted)	–	–
**Current residency**		
Dummy for rural	-0.518*** (0.122)	-0.562*** (0.184)
Intercept	149.757*** (0.25)	162.936*** (0.432)
R2	16,981	10,095
N	0.089	0.067

* OLS Regression with weighted samples. Standard errors in parentheses.

**P < 0.05.

***P < 0.01.

Source: Vélez-Grajales (2016).

The results of the econometric analysis suggest that the increase in height during the second half of the twentieth century is limited, since there was a sustained increase in GDP from 1950 to 1980. When economic performance began to deteriorate in the late 1970s and early 1980s, the average height of the population improved. This highlights that GDP growth does not necessarily move in the same direction or at the same rate as biological living standards. Heights improved for cohorts born in the late 1980s when the economic crisis was at its worst.

**Figure 2:**
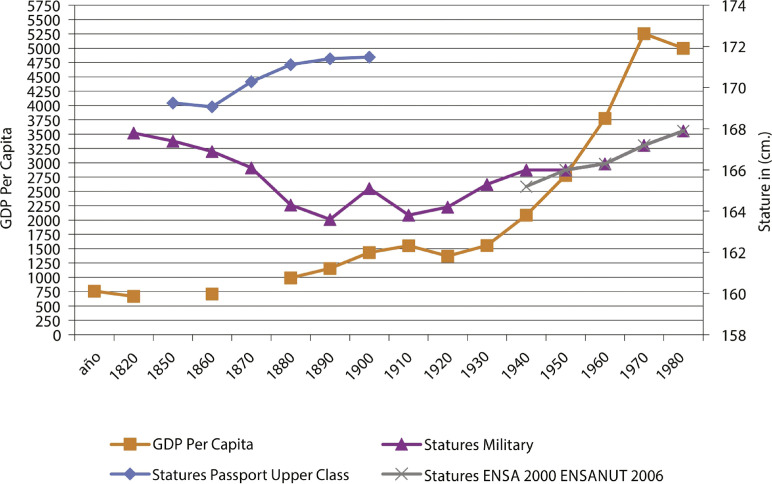
: Graph of men’s heights and GDP per capita measured in international dollars (Geary-Khamis) of 1990 (López-Alonso, Vélez Grajales, 2016)

It is important to analyze these results in the light of a broader context and with respect to case studies that yield more precise results, which help us nuance regional differences by social class among rural populations and gender differences.

As we will see later in this article, most of the studies conducted by the government in the mid-twentieth century to examine the nutritional and health deficiencies of the population focused on rural areas and on the child population in general. In the 1980s, a study was conducted on the urban population to understand intergenerational mobility and its correlation with living conditions (Sandoval, 1985). This study focused on a sample of young men from three different socioeconomic levels. This study looked at mobility patterns in correlation with migration to Ciudad de México in at least three decades before the study was conducted. The findings of this study show that social class was a determinant of height in the early 1980s. Youth whose families had lower incomes were shorter in stature, their families were less educated and larger (p.111). Higher-income youth were taller on average. The difference in height was 12 centimeters between low and high strata, with a difference of 5 centimeters between the middle and high strata (p.176).

At the beginning of the twenty-first century, a study on the nutritional status of Triqui adults, a native ethnic community living in the southwestern part of the state of Oaxaca, showed results that highlight the variety of experiences in the evolution of the living standards of diverse groups, as well as their effects (Ramos Rodríguez, Sandoval Mendoza, 2007). This paper examined the Triqui population in 2002 and compared the results with a similar study conducted in 1940. This study found that height remained very low, especially among women; the community had experienced changes in their eating habits and there was a combination of malnutrition among children and obesity and overweight among adults, especially women. Even though this community lacked public services and its daily activities required considerable energy expenditure, the increase in energy use was less than it had been in previous decades. Their diet now included a high percentage of bottled soda and low-priced industrialized flours (p.265).

The findings of the research in the Triqui community are in line with qualitative research conducted in the state of Morelos, also at the beginning of the twenty-first century, which documents the changes in lifestyles and their repercussions on the health of rural populations that migrated to the city, either to work or eventually to establish permanent residences (Cahuich Campos, 2001). Women who settled in areas on the periphery of cities had less access to fresh food and less time to prepare food for their families (p.130).

It is also important to establish a contrast with the experiences of other countries with which it is possible and relevant to establish comparisons. The case of the heights of the Spanish population is a good comparison for a similar period since there are long-term studies similar to those available for México and above all because this nation had its periods of economic slowdown followed by a recovery. Similar data are available for a similar period, i.e. for those born between 1950 and 1990. In the case of Spaniards, the increase in height for men was approximately six centimeters, while for women it was three centimeters (Candela-Martínez et al., 2022, p.7).

It should be noted that Spaniards had started from a lower height base as the average height of Spanish men in 1854 was 162.29cm (Martínez-Carrión, María-Dolores, 2017), while that of Mexicans was 167cm (López-Alonso, 2015). Another relevant source of comparison is that of Mexican populations that migrated to the United States. Mexican-American children had a growth rate that was converging with that of their peers of Anglo-Saxon origin for cohorts in the 1970s and 1980s (Martorell, Mendoza, Castillo, 1989; Delajara, Rodríguez-Segura, 2010). The study of Guatemalan Mayan children who migrated as refugees to the United States in the 1970s and 1980s compared to their peers who were born and raised in the United States and those who remained in Guatemala also offers a revealing perspective on the possibilities of human plasticity. For children who migrated to the United States, the difference can be five centimeters and ten centimeters for those born in the United States. In this case, there was a substantial increase in height (Bogin, Loucky 1997; Bogin, 2001). Economic improvement opens up the possibility of improving the biological standards of living of populations. An increase of 2.5 centimeters in the Mexican male population at a time when there was economic growth suggests a missed opportunity for living standards. Subsequent studies have confirmed the epigenetic and environmental determinants of human plasticity and its extent (Niere et al., 20 mayo 2020; Bogin, Varela Silva, Rios, 2007; Schefler, Bogin, Hermanussen, 2020).

The causes of this relative stagnation in the height of Mexicans in the second half of the twentieth century are due to high levels of poverty and inequality in the distribution of wealth in the population. Inequality can be caused by deficiencies in various areas. In previous studies, educational inequality has been analyzed (López-Alonso, Vélez-Grajales, 2019, p.15-17). These show that there has been an educational disadvantage. Lower-income sectors are less likely to increase their average schooling, and this is correlated with reduced height. This correlation highlights the vicious circle of poverty. In this study, we will look at the effects of nutrition.

## Nutrition and biological living standards

### Ideas about nutrition

Current thinking on nutrition has been changing, and with it the hypotheses and assumptions that have been made when studies have been carried out. Since the mid-nineteenth century, under the ideas of positivism and social Darwinism, the Mexican population was considered to be flawed in origin due to cultural influences considered primitive and therefore inadequate in terms of best practices for good health and nutrition. These schools of thought can be used to trace the origin of the study of nutrition from a scientific perspective in México. They proposed that the population had to be literally cleansed through miscegenation and adopt ways of life of the Western world in order to improve their living standards (Vargas Domínguez, 2011, 2018, 2019).

During this period, several studies were carried out to adapt the knowledge that emerged around the science of nutrition to the Mexican case (Vargas Domínguez, 2011). It can be argued that Manuel Gamio’s work was a bridge between the work of the Porfirian “hygienists” and post-revolutionary policies. In the 1930s, Manuel Gamio conducted studies on the nutritional state of the marginalized population. The approach was strongly influenced by eugenics theories, with the hypothesis that the indigenous population lived in marginalization due to a kind of racial inferiority, and to bring these groups out of their marginalization, it was necessary to change their living habits, including improving their nutrition. Traditional nutrition was considered deficient because it lacked the proper balance of nutrients and, together with the unhygienic customs of these population groups, caused the majority of the population to suffer from degeneration. During Porfirio Díaz’s term in office, studies were carried out on how to design a balanced diet for the population, taking into account the availability of food in the country.

Gamio’s initiative was not the only one. There were other efforts to improve the nutrition of the Mexican population through social nutrition, as promoted by Doctor Francisco de Paula y Miranda at the National Institute of Nutrition. Social nutritional science sought to approach the issue from a population perspective and as part of a socioeconomic problem, which attempted to improve the quality of life of Mexicans through changes in their diet (Vargas Domínguez, 2019, p.513). In addition to providing courses on food to educate the population to have a better diet, efforts were also made to conduct food surveys. Thereafter, nutrition as a scientific discipline was oriented towards the study of the relationship between food quality and diseases and, as a consequence of this change, the National Institute of Nutrition lost its funding. Nutrition studies continued, but with a “clinical orientation” from the Hospital of Nutrition Diseases, leaving aside the social aspect. This institution was renamed the National Institute of Nutrition under the direction of Doctor Salvador Zubirán (INNSZ) (Vargas Domínguez, 2019, p.531, 542).

It should be added that the advance of nutritional science research was accompanied by the support of the Mexican government to design public policies aimed at improving the living conditions of the Mexican population following what was considered an improvement at that time. During the second half of the twentieth century, clinical research, food biochemistry, and farm productivity converged in the development of public policies aimed at improving welfare levels and alleviating poverty.

### Data sources

An analysis of the surveys and research work carried out by INNSZ during the second half of the twentieth century gives us an idea of how the eating habits of the Mexican population changed as the process of adopting industrialized foods spread throughout the country, and in parallel with changes in lifestyles, urban growth, technological transformation, and production processes. For this purpose, three main sources are examined: the nutritional survey carried out between 1958 and 1962 (México, 1965); the 1979 national food survey (México, 1982); and the study *La nutrición en México y la transición epidemiológica* (Chávez et al., 1993).

It is important to examine the quantitative and qualitative results presented by each work in light of the political, economic, and social context in which they were written. It is important to note that Doctor Adolfo Chávez played a relevant role in the three studies under review; this fact suggests that, even though the results of the three studies are not entirely comparable, and the data they generated do not allow for the construction of a time series, there was continuity in the lines of research. However, the analysis of these three studies as a whole does allow for a qualitative analysis that highlights the causes of the progression of the problem of increasing malnutrition in the Mexican population during the second half of the twentieth century. However, we should not lose sight of the fact that the studies were conducted by researchers who were up to date in their knowledge of the most appropriate research methodologies for this type of study and used the best analytical tools available to them. At the same time, it is important to understand that the focus of each study was informed and constrained by the priorities of the institutions that funded them, even though they were all government agencies. The different orientations reflected the focus and objective of each of the national development programs. Let’s see what these programs were.

### Nutritional surveys in Mexico, 1958-1962

This series of 29 surveys was conducted in 16 major areas of the country with the following objectives: “1. to lay the foundation for planning applied nutrition programs; 2. to facilitate an adequate training program for personnel; 3. to guide future research; and 4. to facilitate the establishment of a national food policy.” The surveys were conducted mainly among the rural and semi-rural population with the notion that “within them the indigenous groups predominate to a certain extent and are undoubtedly the poorest” (México, 1965, p.334).

The Mexican population in the early 1960s was mostly urban. Interestingly, these studies focused on communities that no longer reflected the majority of the population. Notably, it is recognized that the indigenous communities were the poorest. These studies also highlight that “corn is the basis of the diet” and agree with the assertion of Doctor P.D. Martinez who “calls ‘corn man’ (*hombres de maíz*) a person who consumes more than half of his calories from this cereal and consequently suffers from many diseases, has high mortality, has low work performance and practically does not consume other goods and services.” The results emphasize “that pre-Hispanic indigenous foods continue to be consumed mainly in rural areas and that in more than 400 years of contact with other cultures the basis of food has not changed.” It is not clear from the text whether this continuity is good or bad. The consumption of beans, fruits and roots of pre-Hispanic origin is mentioned. As the purchasing power of families increases, we see that there is an increase in the consumption of non-traditional foods, among which were “sugar and limited quantities of meat and other products.” The study explains that consumption patterns are influenced by both cultural and economic factors and that it was observed that there were sought-after and esteemed foods such as meat “and other foods such as pasta, rice etc.” (México, 1965, p.335).

In this study, three types of diet were classified: diet “A”, of indigenous type; diet “B”, of mestizo type typical of semi-rural communities where pasta and rice are included; and diet “C”, with more influence from other cultures with more variety of fruit and vegetables, breakfast with fruit juice, bread and pasta of very different types and dessert at lunch. The summary explains that “[a]ll the diets studied correspond to one of the types or to intermediate situations; moreover, it is possible to argue that there is a marked tendency to evolve along these patterns” (México, 1965, p.336). This clarification suggests that it was considered a situation of dietary improvement to be able to consume foods influenced by other cultures.

The study claims that the poor nutrition of the rural population was due to low or no dietary protein intake. The second most important issue mentioned in the study is the lack of vitamins and minerals in the diet as a cause of nutritional deficiencies. The reason for this is explained by the insufficient income of most families in rural México.

An examination of the surveys and the aspects highlighted in the conclusions of the study shows that the institutional concern was about the state of malnutrition of the population, especially the rural population and with greater emphasis on the child population. The study warned: “Undoubtedly, children suffer the most with a poor, limited and monotonous diet; they are not given the amount they require and the concentration of nutrients in their food is low, especially in protein” (México, 1965, p.339). At the end of the study, recommendations are offered for public policies aimed at improving the nutritional status of the population by offering guidance in the communities. They warned that it was necessary to create “general programs of economic and social development ... to achieve joint action by agencies concerned with nutrition problems and encompassing research, personnel training, technical advice and applied work” (p.341). It remained to be seen how the applied work would be able to bring about change. The objective of studying the child population in order to better serve them responds in part to the fact that at that time the Mexican population was quite young, therefore, improving the health of the youngest was to ensure a more productive society in the future. This objective was very prominent in the presidency of Adolfo López Mateos (1958-1964), with the creation of the National Institute for the Protection of Children (Instituto Nacional de Protección a la Infancia) and the expansion of the school breakfast program (Sefchovich Wasongarz, 2008, p.350).

**Figure 3:**
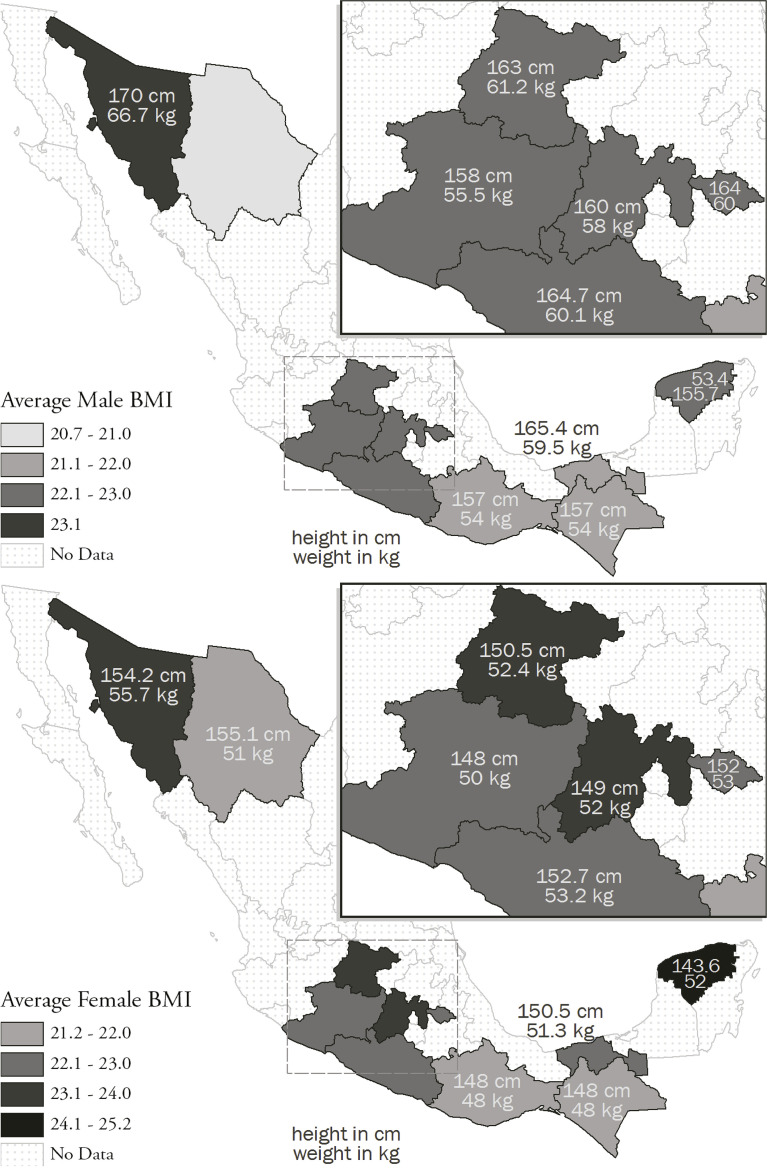
: Body mass index (México, 1965)

There are two other factors worth highlighting from this study. The first is the average of this rural population was not emaciated. A body mass index analysis of the adult population suggests that people were not underweight despite all their nutritional deficiencies (see Figure 3). The second is that foods with influences from other cultures such as sugar and pasta are mentioned as “sought after and esteemed.” The final report pays little attention to this aspect, which will become very relevant as the years go by. As migration to urban centers and to the United States continues and these families increase their income levels, they modify their consumption patterns to something they see as “evolving.” This process can be seen in the source that we will examine below.

### National Company of Popular Subsistence and nutrition policy and public food supply

It is important to note that 17 years elapsed between the development of the two surveys analyzed in this section. The design of the two surveys is quite inconsistent, even though they were designed by the same institute and the same team of specialists. The lack of continuity reflects the food production and supply policies during the six-year terms of Gustavo Díaz Ordaz (1964-1970), Luis Echeverría Álvarez (1970-1976) and José López Portillo (1976-1982). These policies responded to problems that had been brewing for decades. The economic growth and industrialization of the post-revolutionary period were financed by growth in the agricultural sector. This model favored the agro-export sector while marginalizing small producers, communal landholders, and salaried agricultural workers. Many of these rural workers migrated to the cities in search of employment opportunities and expanded the urban working classes. In the 1950s, discontent began to arise due to food shortages and scarcity. The government sought to contain this social conflict through direct intervention in food production, storage, and distribution. Thus, the National Company of Popular Subsistence (Compañía Nacional de Subsistencias Populares, CONASUPO) was created (Pedroza Ortega, 2020). This state-owned enterprise was the central axis of many of the policies related to the “modification in agricultural production sponsored by the Green Revolution, the accelerated incorporation of processed and packaged foods in the consumption pattern” as well as the relationship that the State had with the institutes in charge of the study of nutrition and their collection of information on the different diets in the countryside and the city (Pedroza Ortega, 2020). Thus, through the development of CONASUPO, it can be inferred why the data sources are so fragmentary and why there was a data gap for 17 years.

From the outset, the government’s interest was to respond to a social and economic problem that would legitimize the political system and its revolutionary discourse of social justice. The idea was to produce food in sufficient quantity and at prices that the working classes could afford. If it could also continue with the process of modernization and industrial development, all the better. It was also important to maintain a healthy relationship with the business classes that had been able to grow with the help of government support. It was during this period that there was an important boom in the soda industry, industrial wheat and corn flour, and canned products (Pedroza Ortega, 2020). Each administration had a plan for CONASUPO to meet its objectives since food production and supply problems were far from being solved. Each president had his own central theme: during the six-year term of Díaz Ordaz (1964-1970) the priority was storage and the creation of food distribution networks; the six-year term of Echeverría Álvarez focused on agricultural productivity and the improvement of rural living standards. The six-year term of López Portillo (1976-1982) was confronted with the fact that the country’s economic situation was deteriorating and there was an agri-food crisis. Its focus was on meeting the basic needs of the majority of the population (Pedroza Ortega, 2020, p.254). The brief economic boom generated by the increase in oil production injected resources to address the agrifood crisis. Thus, the Mexican Food System (Sistema Alimentario Mexicano, SAM) and the General Coordination of the National Plan for Depressed Areas and Marginalized Groups (Coordinación General del Plan Nacional de Zonas Deprimidas y Grupo Marginadas, COPLAMAR) were created to work jointly with CONASUPO. The 1979 National Nutrition Survey was carried out against this backdrop.

### National Food Survey, 1979

During the period of the López Portillo government (1976-1982) there was an interest in improving agricultural productivity and guaranteeing food security for the population, hence the creation of the Mexican Food System (SAM). This program was aimed at combating malnutrition and food problems among the country’s population. As part of the activities aimed at achieving this objective, a National Food Survey was conducted in rural areas, coordinated by the INNSZ with the participation of the National Indigenous Institute (Instituto Nacional Indigenista, INI) and the Coordinated Health Services in the states, as well as the Ministry of Health and Assistance. This survey focused on collecting data to measure food consumption per person, per day. The sampling was different from the first survey. The data first published in 1982 did not include an exhaustive analysis of all the information collected but was then considered “the first chapter of information analysis” where nutrient inputs and other data could be calculated. The tables by states and maps showing the states and the different nutritional zones covered by each state are included (see Table 3).

**Table 3:** : National Nutrition Survey, 1979

Zones*	**1**	**2**	**3**	**4**	**5**	**6**	**7**	**8**	**9**	**10**	**11**	**12**	**13**	**14**	**15**	**16**	**17**	**18**	**19**
**Corn**	77.2	78.67	172.14	329.51	281.51	259.48	377.11	344.97	292.99	360.9	391.77	292.63	273.71	411.02	325.39	378.78	333.84	218.4	369.51
**Wheat**	99.85	138.67	214.61	118.43	53.53	33.37	10.5	2.31	8.1	3.33	2.72	1.67	0.19	1.5	0.71	26.95	0.12	0.06	0.92
**Bread**	14.91	22.89	17.17	16.41	23.01	37.78	35.68	27.52	40.72	45.56	50.87	68.9	23.2	33.3	46.86	30.08	13.84	42.52	51.5
**Pasta**	6.73	13.31	16.02	23.49	22.78	15.4	13.43	18.43	11.79	14.1	12.81	15.37	6.52	19.52	12.9	6.39	6	21.49	5.1
**Rice**	10.06	20.86	10.41	7.73	12.32	16.68	7.37	11.64	10.53	10.69	15.57	8.87	10.4	9.67	20.44	9.33	8.23	41.43	10.45
**Flour**	1.04	0.81	1.15	11.02	0	3.95	0.08	0.51	1.37	2.58	0.25	0.88	0	0.29	0.1	1.1	0	0.01	0.63
**Beans**	22.53	33.18	14.08	49.83	45.62	32.27	34.97	35.24	31.22	36.26	37.54	25.03	19.47	38.52	29.98	34.06	38.18	16.4	37.18
**Roots**	43.29	52.18	46.16	49.64	38.67	40.56	33.99	22.49	20.49	18.64	21.16	14.91	11.11	19.25	10.89	12.57	15.93	32.89	6.76
**Green Leaves**	6.29	47.45	17.33	15.53	8.94	21.04	19.13	21.39	16.44	17.75	8.31	18.44	3.37	9.94	7.67	25.27	8.29	10.87	1.43
**Chili**	2.73	4.55	5.04	17.79	8.72	4.83	1.79	5.95	8.66	6.57	11.58	6.42	4.99	10.04	6.43	4.51	1.71	1.15	1.47
**Tomatoes**	10.83	32.8	23.04	29.35	43.74	44.05	44.44	18.55	40.66	35.65	34.37	42.91	15.07	38.22	41.56	24.06	11.87	17.33	11.31
**Bananas**	43.37	34.04	41.75	32.96	31.87	50.21	55.47	16.98	48.82	37.68	63.79	41.27	22.45	54.3	56.99	23.49	20.89	41.53	32.81
**Citrus Fruits**	1264	126.9	118	8.34	8.06	58.43	181.09	0.77	41.38	15.62	14.35	16.19	13.32	4.66	22.47	11.38	11.21	18.96	25.39
**Other Fruits**	1.65	0	2.34	0	1.15	0.09	0.03	0	1.27	0.23	0.68	0.13	0.16	0.4	0.03	1.24	0	0	0.3
**Milk**	239.21	160.53	132.08	153.4	130.6	157.6	252.97	154.27	288.33	192.01	36.09	121.49	115.57	48.49	138.32	7.13	8.93	83.19	19.36
**Cheese**	9.33	8.88	23.85	8.58	2.81	13.01	22.71	6.06	9.69	5.08	1.3	3.14	6.66	1.97	5.96	1.82	3.19	3.82	1.36
**Meat**	55.38	100.13	83.19	37.55	60.69	74.67	110.71	26.45	56.47	40.06	47.95	64.95	40.75	25.14	86.58	36.18	61.09	197.5	68.31
**Egg**	29.99	46.33	50.83	31.35	46.12	48.1	45.32	21.31	19.37	18.09	24.42	19.99	13.61	18.48	28.75	16.24	24.2	23.55	37.84
**Sugar**	24.68	53.11	45.65	68.26	35.55	48.96	46.61	22.69	38.83	34.01	47.19	36.88	20.29	25.04	63.83	38.17	38.81	88.33	39.45
**Soda**	133.44	46.61	128.95	137.25	223	242.33	106.08	64.98	98.46	69.34	95.15	50.71	34.59	18.51	73.4	31.38	13.73	34.33	128.56
**Pulque**	0.25	0	0	0.52	5.75	0.8	1.09	0	0.23	14.83	8.62	51.72	0.66	133.16	0.76	1.56	0	0.23	0.44
**Beer**	5.52	8.93	11.78	0.19	3.13	29.65	3.57	3.57	13.31	1.65	3.6	2.97	2.27	0.47	9.1	4.26	5.46	2.87	6.65
**Fat**	50.49	54.05	58.34	47.86	46.02	49.67	38.32	21.93	23.73	22.81	25.12	20.35	13.08	21.9	35.67	8.28	11.97	34.45	17.1
**Salt**	3.93	10.22	17.41	10.58	14.46	8.51	13.22	8.11	10	8.07	6.38	5.11	9.49	7.21	8.2	7.88	6.02	8.14	4.56

*For the selection sample, the geoeconomic regions proposed by Bassols Batalla (1964) were taken. Nutritional zones comprise several geoeconomics regions and complete or fractional states. Zone 1: Baja California Norte, Coahuila, Chihuahua, Sonora, Tamaulipas; Zone 2: Baja California Norte, Baja California Sur; Zone 3: Sonora, Sinaloa; Zone 4: Chihuahua, Durango, Nayarit; Zone 5: Coahuila, Durango, Nuevo León; Zone 6: Tamaulipas; Zone 7: Sinaloa, Nayarit; Zone 8: Zacatecas, San Luis Potosí; Zone 9: Zacatecas, Aguascalientes, Jalisco; Zone 10: Guanajuato, San Luis Potosí, Michoacán, México, Querétaro; Zone 11: San Luis Potosí, Hidalgo, Veracruz, Puebla, Querétaro; Zone 12: Hidalgo, México, Tlaxcala; Zone 13: Morelos, Colima, Michoacán, México, Guerrero; Zone 14: Tlaxcala, Puebla; Zone 15: Veracruz, Oaxaca; Zone 16: Puebla, Guerrero, Oaxaca; Zone 17: Chiapas; Zone 18: Veracruz, Tabasco, Chiapas y Campeche; Zone19: Campeche, Yucatán, Quintana Roo.

Source: México, 1982.

It should be noted that there was an interesting change in the list of foods that made up the basic diet being surveyed. Bread, pasta, sugar flour and soda are included. It is also worth noting that there are regional deficiencies in the degree of adoption of more “modern” foods. Unlike the previous census, these foods were already part of the diet. The approach was more macroeconomic, and less emphasis was given to nutrition quality. The increase in the intake of soda, pasta and flour is not questioned. It gives the impression that there was still an interest in combating malnutrition rather than an interest in understanding the nature of malnutrition. The emphasis was on making sure that caloric intake was sufficient and not on the quality of the diet. The economic crisis also had an impact, forcing budget cuts that reduced the frequency and scope of the surveys. The reflection of the specialists was that “[t]he crisis and the end of the government period provoked, once again, the dismantling of the program and the cancellation of the generation of epidemiological information on the levels and trends of malnutrition in the country” (Avila-Curiel et al., 1993, p.660).

### Measurement in times of economic crisis, *Nutrition in Mexico and the epidemiological transition*

In times of austerity in government spending, resources for the elaboration of surveys were allocated to the study of health. In 1986, the National Health Survey System was created by the National Institute of Public Health. In the early 1990s it was recognized that the economic conditions of crisis that México suffered during most of the 1980s were not good for research on the quality of nutrition of the population. Due to the crisis, there were no longer resources to provide continuity to public policies for food improvement that reflected both the changing needs of the population and what nutrition research was being able to recommend. Data collection was insufficient. According to the specialists:

From 1982-1988, there was an information gap in terms of the epidemiological surveillance of nutrition. Infant and preschool mortality statistics were no longer published and there was a five-year delay in their publication. There was insufficient support for research on the nutritional conditions of the population, so it was considerably reduced (Avila-Curiel et al., 1993, p.661).

In the early 1990s, a study was also published that made a diagnosis of what had been happening since the 1950s in terms of dietary changes and that by the time of publication had reached a turning point. Since 1981, there has been an accelerated process of change in morbidity and mortality, and chronic non-communicable diseases, especially diabetes, hypertension, thromboembolism, and various types of heart disease have also begun to increase. It was in this “year that mortality from heart disease clearly surpassed that from respiratory infections.” This phenomenon was called an “epidemiological trap” because several infectious diseases were still present, including childhood malnutrition-infection, with the addition of chronic diseases such as obesity, diabetes, and atherosclerosis in adults (Chávez et al., 1993, p.77). The study points out that there were economic, food and health reasons for this phenomenon. The economic causes were generated by the crisis that had afflicted the country since the beginning of the 1980s.

Regarding the causes related to food, it is argued that there was a change in the diet from being composed mostly of corn and beans (approximately 70%) to other foods that were not “better,” i.e. pasta soups, pastries, fried foods, or bread. Since then, it has been warned that this diet “in the long run will no longer provide major health benefits. Children will survive in greater numbers, but not with better physical, mental, or social quality” (Chávez et al., 1993, p.78). There are three situations that exemplify what many low-income households could be experiencing at this time when malnutrition coincided with chronic diseases in the same family:

1. Rural-urban migration meant that a rural child who had already been malnourished when he was young had a stable job and then ate without measure the festive foods of his town, such as meat, pork rinds and carnitas, and easily became obese.
2. In the same family, a child may be malnourished because he or she was no longer breastfed, and the parents did not have the knowledge or resources to feed the infant adequately while the parents overeat and eat incorrectly.
3. A person who decides to follow “the ‘modern and prestigious’ pattern of eating ready-to-use foods that are advertised in the media and that were pleasant to taste, and therefore could have a diet so incorrect that it led to various deficiencies with various chronic pathological alterations” (Chávez et al., 1993, p.79).

With regard to the reasons related to health, the study emphasizes that as a result of the economic crisis, government spending on health had decreased substantially. Although the fight against communicable diseases was important, as well as vaccination and maternal and child care, which were the priorities of the Ministry of Health, due to lack of budget, there were lags in several areas such as preventive health programs.

It was warned that in México the epidemiological transition in the country was more of an “epidemiological trap.” This warned that there would be a sharp increase in morbidity from various chronic diseases to the extent of a true epidemic. The researchers concluded the study by asserting that “a more decisive social participation was required, in which the problem of food and bad living habits would be confronted with a less economic and less political criterion, but rather with a more social and more human one” (Chávez et al., 1993, p.84). The small print run and limited distribution of this study reveals that, although there was continuity in the research, the findings were not incorporated into nationwide initiatives such as the national health survey system mentioned above. This study is a convincing proof that the experts in the field, despite the need to create more complete statistics, had enough information and knowledge to diagnose the problem that has continued to grow, keeping in force the public policy recommendations formulated in this document (México, 2020, p.11-12).

The data and results presented in the sources examined do not provide data that can be incorporated into an econometric exercise for a more accurate measurement of how much the quality of the diet influenced the final height of the population. Nutrition surveys can give us an approach of the quality and quantity of the diet, but not what actually happens on a daily basis. We know that unlike height, adult weight can increase or decrease over time, and this is more related to eating habits and lifestyles than to the quality of nutrition during the first 20 years of life. Quantifying diet quality is complicated; however, it is important to incorporate this information into the set of determinants of the biological standards of living of the Mexican population. Even if it has only been possible to observe how eating habits have been changing, this is fundamental to understanding the increase in the incidence of chronic degenerative diseases.

## Final considerations

In this paper we have used the evolution of the average height of the population as an indicator of the evolution of biological standards of living and have explained why it is a reliable indicator and how it allows comparisons with other human populations. We found that there was an increase of 2cm and 2.5cm for males and females respectively who were born and grew up in the second half of the twentieth century. Although it can be argued that this is an improvement in the living standards of the population, given that life expectancy at birth between 1950 and 1990 increased from 49.7 years to 67.2 years, the improvement in average height is low with respect to the possibilities of human plasticity given the economic, political, and environmental circumstances in that period. Social programs were launched to provide health and education services and to promote better nutrition for children. This increase is moderate when compared to other countries or to Mexican migrant populations with similar characteristics. The Mexican population did not have an even greater increase in its average height due to inequality in the distribution of resources. Social programs did not reach all those who needed them, and small farmers produced less. All this kept the bulk of the population in poverty and marginalization. In addition, there was a change in eating habits.

The government tried to improve the population’s standard of living. However, the way in which resources were allocated to fulfill this purpose had its limitations, in addition to the aggravating factor of demographic dynamics. Physicians and specialists in nutrition and public health followed up on the nutritional status of the population by conducting studies focused on understanding the situation of key segments of the population such as children and the rural population. The diagnoses and public policy recommendations were correct: it was necessary to increase the intake of animal protein and increase the amount and variety of fruits and vegetables in the diet. Unfortunately, these recommendations were not a central focus of the national development plans. The various governments of this period chose to focus more on food production and distribution with the objective of increasing coverage rather than on the quality of the food distributed. For its part, the food industry used advertising to promote the consumption of its products, selling an image of modernity and social improvement. This type of advertising was well received by the rapidly growing urban population, whose changing lifestyles favored the consumption of industrialized foods.

The phenomenon of change in eating habits has occurred in other countries of the world, both rich and poor. In the case of México, what is relevant is that this change took place at a time and under circumstances that limited the scope of the economic and public health improvements that were being experienced. This paper seeks to contribute to the study of living standards and inequality from the fields of public health history and economic history.
